# Plasticity in Dendroclimatic Response across the Distribution Range of Aleppo Pine (*Pinus halepensis*)

**DOI:** 10.1371/journal.pone.0083550

**Published:** 2013-12-31

**Authors:** Martin de Luis, Katarina Čufar, Alfredo Di Filippo, Klemen Novak, Andreas Papadopoulos, Gianluca Piovesan, Cyrille B. K. Rathgeber, José Raventós, Miguel Angel Saz, Kevin T. Smith

**Affiliations:** 1 Departamento de Geografía y Ordenación del Territorio, Universidad de Zaragoza, Zaragoza, Spain; 2 Instituto de Investigación en Ciencias Ambientales (IUCA), Universidad de Zaragoza, Zaragoza, Spain; 3 Department of Wood Science and Technology, University of Ljubljana, Ljubljana, Slovenia; 4 DendrologyLab (DAFNE), Università Degli Studi della Tuscia, Viterbo, Italy; 5 Department of Forestry and Natural Environment Management, Technological Education Institute of Lamia, Karpenissi, Greece; 6 Laboratoire d'Etude des Ressources Forèt-Bois (LERFoB), Centre INRA de Nancy, Champenoux, France; 7 Departamento de Ecología, Universidad de Alicante, San Vicente del Raspeig, Spain; 8 Northern Research Station, USDA Forest Service, Durham, North Carolina, United States of America; DOE Pacific Northwest National Laboratory, United States of America

## Abstract

We investigated the variability of the climate-growth relationship of Aleppo pine across its distribution range in the Mediterranean Basin. We constructed a network of tree-ring index chronologies from 63 sites across the region. Correlation function analysis identified the relationships of tree-ring index to climate factors for each site. We also estimated the dominant climatic gradients of the region using principal component analysis of monthly, seasonal, and annual mean temperature and total precipitation from 1,068 climatic gridpoints. Variation in ring width index was primarily related to precipitation and secondarily to temperature. However, we found that the dendroclimatic relationship depended on the position of the site along the climatic gradient. In the southern part of the distribution range, where temperature was generally higher and precipitation lower than the regional average, reduced growth was also associated with warm and dry conditions. In the northern part, where the average temperature was lower and the precipitation more abundant than the regional average, reduced growth was associated with cool conditions. Thus, our study highlights the substantial plasticity of Aleppo pine in response to different climatic conditions. These results do not resolve the source of response variability as being due to either genetic variation in provenance, to phenotypic plasticity, or a combination of factors. However, as current growth responses to inter-annual climate variability vary spatially across existing climate gradients, future climate-growth relationships will also likely be determined by differential adaptation and/or acclimation responses to spatial climatic variation. The contribution of local adaptation and/or phenotypic plasticity across populations to the persistence of species under global warming could be decisive for prediction of climate change impacts across populations. In this sense, a more complex forest dynamics modeling approach that includes the contribution of genetic variation and phenotypic plasticity can improve the reliability of the ecological inferences derived from the climate-growth relationships.

## Introduction

Climate strongly influences the geographical distribution of plant species in terrestrial ecosystems [1], [2]. As local temperature and water availability change with broader changes in climate, biodiversity, species distribution, and growth rates are expected to change accordingly [3], [4], [5]. However, the impact of climate change may vary greatly depending on the ability of species to acclimate or to adapt to future climate conditions [6].

Plant species can adjust to new environmental conditions through local adaptation or phenotypic plasticity [6]. Local adaptation may imply genetic differentiation among populations as a consequence of differential selection pressures and/or population isolation [7]. Phenotypic plasticity, defined as the range of phenotypes that a single genotype can express as a function of its environment, can also be a crucial factor for plant response to rapid climate change [6], [8]. Heterogeneous responses of species to climate variability across their range are directly connected to phenomena of local adaptation and phenotypic plasticity and are the basis of potential adaptability to future climate conditions. Detailed knowledge of the relationship between climate and growth across the range of distribution of species is essential to predict and mitigate the effects of climate change.

Climate change is especially rapid and extreme in the Mediterranean basin [9], [10], [11]. However, due to the transitional nature of the Mediterranean climate (which ranges from near-desert to temperate regimes), Mediterranean areas contain a great variety of natural conditions which will likely affect the impact of climate change [12], [13], [14].

In the widely diverse set of climatic conditions contained in the Mediterranean basin, pines are by far the most widespread genus covering approximately 5% of the total land area and 25% of the forested area. The most common species is the Aleppo pine (*Pinus halepensis* Mill.) occupying large areas of the western Mediterranean as well as occurring in the eastern Mediterranean [15], [16]. The ecology and biogeography of Aleppo pine is well-researched [17], [18]. The dendroclimatology of Aleppo pine has been investigated at individual locations within the Mediterranean basin [19], [20], [21], [22], [23], [24], [25], [26], [27], [28], [29] confirming that Aleppo pine is sensitive to climate variation. However, a comprehensive dendroclimatic analysis across the distribution range of Aleppo pine is lacking.

In this paper, we investigate the variability of the climate-growth relationships of Aleppo pine using a dendroclimatic network composed of high-resolution climatic and dendrochronology databases comprising the complete climatic gradient across the Mediterranean Basin. Our objectives were to a) characterize the climatic variability within the range of Aleppo pine; b) relate high frequency variation in tree-ring width to monthly, seasonal, and annual temperature and precipitation; c) determine the dependence of the dendroclimatic relationships to underlying climatic patterns.

## Methods

### Ethics Statement

All sampling sites were located in public forests. The necessary permits for field sampling were issued by the forestry authorities at regional and local level. The locations were not protected areas, and the field studies did not involve endangered or protected species.

### Locations and climatic data

**Figure 1:**
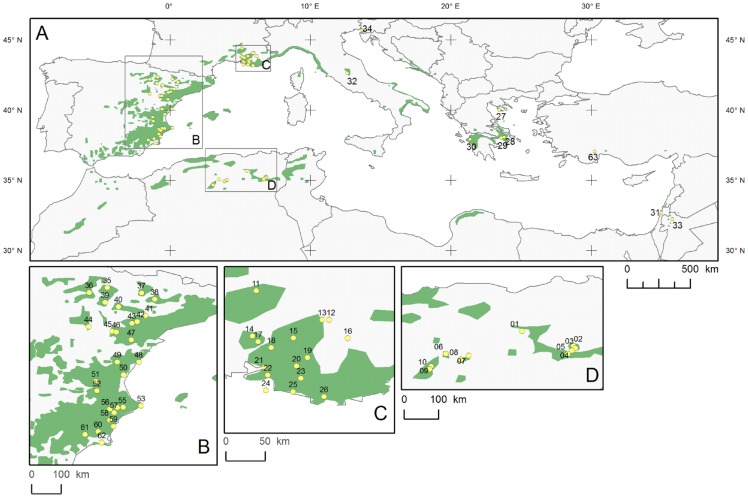
Map of the Mediterranean Basin and the distribution of Aleppo pine (A); details in B, C, D (bottom).

The distribution map for Aleppo pine (Fig. 1) based on an earlier map [30] was obtained from the European Forest Genetic Resources Programme (EUFORGEN http://www.euforgen.org/distribution_maps.html). Climatic time series data for 1,068 spatial grid points within the distribution range for the period 1901–2000 were obtained from the Climatic Research Unit (CRU) of the University of East Anglia. For the western part (Spain, Algeria, France, Italy, Slovenia, Greece, and Turkey), monthly, seasonal, and annual mean temperatures and total precipitation were provided by the CRU TS 1.2 dataset (10-minute resolution) [31]. For the portion of the distribution located east of the coverage of CRU TS 1.2, we used the CRU TS 2.1 dataset (0.5° grid resolution) [32]. To characterize the spatial variability of the climate across the distribution area of Aleppo pine, while minimizing the effect of outlier values, we determined the central 95% range for each variable, defined as the values occurring between the 2.5 and 97.5 ranked percentiles.

The dendrochronological network consisted of newly collected and archived tree-ring width series from 63 Aleppo pine sites (Table 1), between 32.23° and 45.67°N latitude, 1.41°W and 36.17°E longitude, and 15 to 1650 m a.s.l. (Fig. 1). To determine the adequacy of the network to describe the range of dendroclimatic responses, climatic data for the CRU gridpoint closest to each site were compared with those of the Aleppo pine distribution as a whole.

**Table 1:** Study sites.

N°	Site code	Site	Country	Lat	Long	Elevation	Trees	Samples	Tree-rings	EPS>0.85	MS (res)	MC (res)
**1**	**BOU**	Bout10(***)	Algeria	35.65	5.5	1200	11	26	2463	1893–1988	0.3	0.43
**2**	**CHE**	Chelia 9(***)	Algeria	35.25	6.85	1650	11	32	4330	1819–1988	0.25	0.68
**3**	**BEZ**	Bezez 8(***)	Algeria	35.2	6.9	1300	12	34	3558	1864–1988	0.4	0.67
**4**	**TZI**	Tizi Tmellet 6(***)	Algeria	35.13	6.8	1450	10	24	3264	1831–1988	0.47	0.74
**5**	**RAS**	Ras fourar 7(***)	Algeria	35.05	6.7	1200	9	27	3342	1835–1987	0.47	0.72
**6**	**SAH**	Sahari 3(***)	Algeria	35.05	3.5	1060	11	32	3843	1849–1989	0.3	0.6
**7**	**MES**	Messaad 5(***)	Algeria	35	4.1	1100	11	31	3354	1797–1989	0.41	0.75
**8**	**SAF**	Safai 4(***)	Algeria	34.9	3.9	1100	11	28	2925	1851–1989	0.4	0.72
**9**	**SEN**	SenalbaN(***)	Algeria	34.72	3.12	1410	11	33	3322	1858–1988	0.31	0.68
**10**	**SEW**	SenalbaW(***)	Algeria	34.65	3.08	1350	11	33	3451	1859–1990	0.37	0.74
**11**	**NYO**	Nyons(*)	France	44.36	5.16	350	8	14	1171	1906–1993	0.23	0.6
**12**	**MEE**	Les Mées(*)	France	44.03	5.99	600	9	12	941	1912–1994	0.29	0.58
**13**	**PER**	Peruis(*)	France	44.03	5.91	600	8	13	1001	1924–1992	0.18	0.43
**14**	**ROB**	Robion(*)	France	43.84	5.12	300	11	20	1663	1905–1994	0.31	0.57
**15**	**CVI**	Vitrolles(*)	France	43.82	5.58	600	9	12	919	1918–1993	0.28	0.51
**16**	**MOU**	Moustier Sainte-Marie(*)	France	43.82	6.21	750	10	12	1062	1900–1994	0.32	0.57
**17**	**FOO**	Mérindol(*)	France	43.78	5.18	330	9	13	1398	1881–1994	0.35	0.69
**18**	**RDA**	Roque d'antheron(*)	France	43.71	5.33	170	13	19	1645	1896–1994	0.27	0.59
**19**	**RIA**	Rians(*)	France	43.6	5.75	450	10	14	1179	1903–1994	0.32	0.63
**20**	**ROU**	Rousset(*)	France	43.5	5.62	200	6	6	426	1920–1990	0.31	0.71
**21**	**ROG**	Rognac(*)	France	43.48	5.26	190	7	15	1346	1903–1994	0.34	0.56
**22**	**PNM**	Les Pennes Mirabeau(*)	France	43.4	5.29	150	7	14	1050	1910–1993	0.34	0.6
**23**	**AUR**	Auriol(*)	France	43.36	5.67	300	8	11	1040	1901–1993	0.28	0.51
**24**	**MAR**	Marseille(**)	France	43.22	5.27	200	7	21	2664	1833–1973	0.42	0.64
**25**	**CIO**	La Ciotat(*)	France	43.21	5.58	200	12	17	1517	1899–1994	0.31	0.5
**26**	**FAR**	Toulon(*)	France	43.15	5.94	500	9	15	1353	1904–1993	0.23	0.45
**27**	**KAS**	Kassandra	Greece	39.98	23.47	136	10	30	1369	1937–1989	0.27	0.71
**28**	**KSY**	Athens1	Greece	38.05	23.82	260	10	17	1777	1910–2003	0.31	0.48
**29**	**DBG**	Athens2	Greece	38	23.62	180	23	37	3183	1909–2001	0.35	0.37
**30**	**AMA**	Amaliada	Greece	37.9	21.4	88	12	36	1400	1948–1989	0.21	0.64
**31**	**CAR**	Carmel Mountain(*****)	Israel	32.67	35	450	8	9	613	1959–1997	0.27	0.43
**32**	**TER**	Terni	Italy	42.62	12.65	470	33	33	1985	1940–2003	0.21	0.3
**33**	**DIB**	Dibeen(*****)	Jordan	32.23	35.82	867	14	14	819	1936–1993	0.29	0.4
**34**	**SLO**	Krkavce-Dekani	Slovenia	45.67	13.7	50	25	49	2310	1949–2004	0.23	0.38
**35**	**AYE**	Ayerbe (Biel)	Spain	42.32	−0.84	924	19	33	1377	1962–2006	0.23	0.45
**36**	**EBA**	Ejea-Bardenas	Spain	42.17	−1.4	365	7	7	499	1941–2003	0.32	0.56
**37**	**GRA**	El Grado	Spain	42.16	0.2	168	15	30	1506	1950–2006	0.24	0.56
**38**	**EST**	Estopiñan del Castillo	Spain	41.97	0.61	502	14	27	1087	1965–2006	0.38	0.62
**39**	**VLL**	Villanueva de Gállego	Spain	41.88	−0.91	452	15	29	2692	1898–2006	0.4	0.58
**40**	**CAP**	Alcubierre (San Caprasio)	Spain	41.75	−0.5	738	14	27	1516	1942–2006	0.29	0.63
**41**	**FRA**	Fraga	Spain	41.47	0.32	340	14	29	3858	1857–2006	0.43	0.62
**42**	**CAS**	Caspe	Spain	41.29	0.07	166	15	28	3539	1856–2007	0.59	0.68
**43**	**CHI**	Chiprana	Spain	41.24	−0.09	160	7	9	756	1933–2003	0.32	0.45
**44**	**DAR**	Daroca	Spain	41.14	−1.41	937	14	28	1699	1936–2006	0.42	0.83
**45**	**OLI**	Oliete	Spain	40.99	−0.69	530	15	28	1099	1961–2006	0.3	0.5
**46**	**ALL**	Alloza	Spain	40.98	−0.57	595	17	31	2878	1905–2006	0.37	0.69
**47**	**ZOR**	Zorita	Spain	40.74	−0.11	857	15	30	3004	1884–2001	0.4	0.61
**48**	**ORO**	Oropesa	Spain	40.06	0.12	1	15	30	2022	1929–2003	0.32	0.54
**49**	**MON**	Montanejos	Spain	40.06	−0.54	569	16	31	1223	1956–2001	0.39	0.67
**50**	**GIL**	Gilet (Sant Espirit)	Spain	39.67	−0.35	175	14	27	2323	1910–2006	0.65	0.7
**51**	**REQ**	Requena	Spain	39.47	−1.2	721	15	31	3858	1816–2003	0.35	0.48
**52**	**JAL**	Jalance	Spain	39.19	−1.15	571	15	35	3034	1881–2003	0.41	0.65
**53**	**JAV**	Javea	Spain	38.73	0.19	96	15	60	3434	1927–2000	0.35	0.73
**54**	**ALC**	Alcoy (Penaguila)	Spain	38.68	−0.36	674	15	30	3792	1863–2001	0.32	0.51
**55**	**FNT**	Alcoy (Font Roja)	Spain	38.67	−0.54	1022	14	24	1960	1881–2006	0.35	0.61
**56**	**BIA**	Biar	Spain	38.62	−0.77	806	15	29	1798	1930–2001	0.29	0.73
**57**	**MAI**	Maigmo	Spain	38.52	−0.64	845	15	31	2697	1896–2000	0.4	0.65
**58**	**CRE**	Crevillente	Spain	38.29	−0.77	285	12	43	3715	1911–2000	0.45	0.49
**59**	**GUA**	Guardamar	Spain	38.1	−0.65	15	30	77	6047	1916–2006	0.36	0.59
**60**	**FUE**	Fuensanta	Spain	37.94	−1.12	138	14	26	2094	1908–2007	0.44	0.64
**61**	**SEÑ**	Sierra Espuña	Spain	37.86	−1.52	846	16	29	2670	1907–2007	0.25	0.49
**62**	**CAT**	Cartagena	Spain	37.61	−1.01	116	15	27	2039	1921–2007	0.42	0.61
**63**	**BAB**	Bayat Bademleri(****)	Turkey	37.02	30.28	700	15	15	2567	1752–2001	0.21	0.61

DendroDB (Nicault);

ITRDB (Serre-Bachet);

DendroDB (Safar);

ITRDB (Schweingruber),

ITRDB (Touchan),

General information on the 63 study sites in the Aleppo pine network. LAT: Latitude (°N); LON: Longitude (°E); ELE: Elevation (m a.s.l); MS: mean sensitivity; MC: mean correlation between trees.

A T-mode Principal Component Analysis (PCA) [33] was used to summarize the spatial variability of the mean climate conditions across the study area. PCA is a data reduction technique that transforms a large group of variables into a new smaller set of variables called principal components (PC), which are linear combinations of the original variables. These PC were calculated on the correlation matrix of mean monthly temperatures and mean total monthly precipitation for the period 1901–2000. The components were rotated (Varimax) to redistribute the explained variance and to obtain more stable and robust spatial patterns [34]. Selected PC, guided by Kaiser's Rule (eigenvalues >1; [35]), described climatic gradients across the Mediterranean basin were used in subsequent analysis.

### Tree-ring chronology construction

The tree-ring width series were derived from a total of 1634 increment cores from 818 trees (Table 1). Tree selection, core collection, processing, and ring-width measurement were conducted using standard techniques [36], [37]. Ring-width index chronologies for each site in the network were constructed using ARSTAN software version 6.05P [38] to retain the high-frequency variation that would be most sensitive to the climate variables to be tested. First, a negative exponential or linear regression function was fitted to each ring-width series. The residuals from this first detrending were then fitted with a cubic smoothing spline function (50% frequency cut-off of 30 years). The residuals from the second detrending were then fitted with an autoregressive model to reduce the autocorrelation in the model residuals which were then averaged to construct the ring-width residual index chronology (chronology RES in ARSTAN) for each site using the biweight robust mean function. An expressed population signal (EPS) of 0.85 was used as a threshold to determine the reliable part of each index chronology to use in subsequent analysis [39].

### Dendroclimatic relationships across climate gradients

For our first-stage tests, the statistical relationship between monthly, seasonal and annual climate series and annual tree-ring chronologies was assessed individually for each of the 63 sites by correlation function (CF) analysis using the program DendroClim2002 [40]. The RES chronology was the dependent variable while the regressors were the monthly, seasonal and annual mean temperatures and the total precipitation for each 16-month biological year (from the previous September to the current December).

The second-stage test consisted of multiple regression analysis (forward selection) using the CF coefficients calculated in the first stage as dependent variables and the significant principal components (PC) as independent variables that characterize mean climate conditions for each of 63 study sites. In this test, dependence of CF on PC indicates that the correlation of growth to annual, seasonal, and monthly climate variability depended on the underlying climatic conditions. Then, if dendroclimatic relations were found to be climate-dependent, the variation in the climate-growth relationship may be quantified and predicted for the whole distribution area of species by resolving obtained regression models on all 1,068 points for which PC values were calculated.

## Results

### Climate characteristics within the distribution area of Aleppo pine

**Figure 2:**
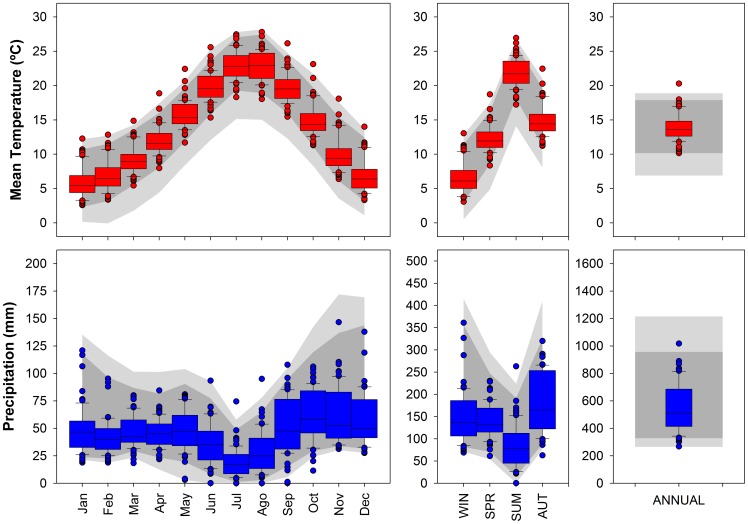
Climate characteristics across the distribution area of Aleppo pine. Dark grey and light grey areas represent climate envelopes defined by the 2.5th–97.5th and the 0.5th–99.5th percentiles, respectively. Box plots show the range of mean monthly air temperatures and total monthly precipitation for the *P. halepensis* dendrochronological network of 63 locations. The central vertical lines indicate median values, boxes enclose the central two quartiles, whiskers indicate the 10th and the 90th percentiles and the dots represent the full range of mean climate values.

The climatic conditions varied widely from near desert to moist temperate. The central 95% of the range (bounded by the 0.025 and 0.975 percentiles) of mean annual temperatures ranged from 10.2°–17.9°C. Winter temperature (average of 7.0°C) ranged from 2.9–11.5°C and summer temperature (average of 22.3°C) ranged from 18.2–25.9°C (Fig. 2). The central 95% range of annual total precipitation (average of 570 mm) across the geographic distribution ranged from 328–957 mm. The seasonal distribution of precipitation varied greatly with winter precipitation comprising about 31% of the total annual precipitation. Among sites, winter precipitation contributed 15–66% of the annual total precipitation. Spring precipitation contributed an average of about 27% of the total annual precipitation with sites ranging from about 12–36%. Autumn precipitation contributed an average of about 30% with a range of about 13–43%. Summer was generally the dry season with an average annual contribution of about 11% of the total precipitation but ranged across sites from less than 0.1% to about 29% of the total annual precipitation (Fig. 2).

**Figure 3:**
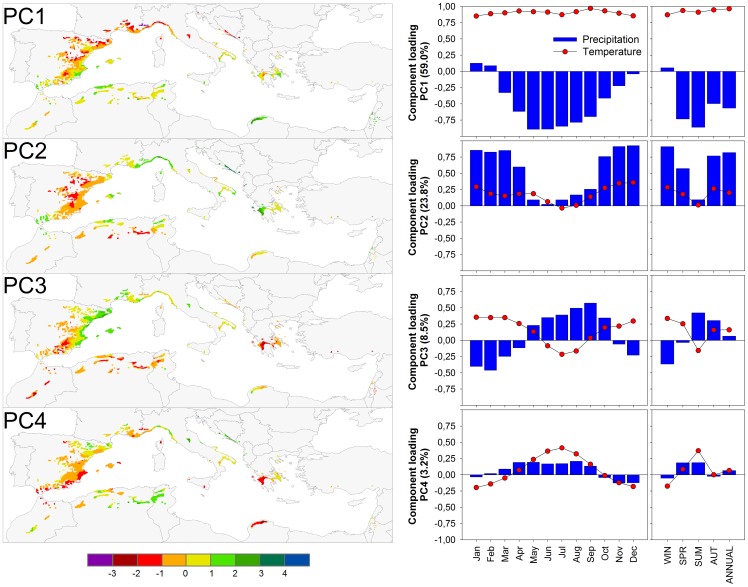
Spatial distribution of the first four significant principal components (PC) reflecting the spatial variability of the mean climate conditions (left). Component loading of each PC against mean monthly, seasonal and annual temperatures and precipitation for the period 1901–2000 (right).

The climate variation across the distribution area of Aleppo pine can be summarized by four significant principal components (PC) which together explain about 95% of the variation. PC1 (containing 59% of the variation) represented the main climatic gradient, from cold and wet conditions to warm and dry (Fig. 3). PC2 (about 24%) related to variation in precipitation (Fig. 3). PC3 (about 8%) represented variation in the seasonal distribution of precipitation from sites where winter is the main precipitation season to sites where the contribution of summer and autumn precipitation to the total annual precipitation increases (Fig. 3). PC4 (about 3%) represented variation in temperature between winter and summer (Fig. 3).

### Dendroclimatic relationships in the Aleppo pine tree-ring network

The basic statistics for 63 local chronologies are shown in Table 1. Mean correlation between trees and mean sensitivity of chronologies varied from 0.30 to 0.83 (average of 0.59) and from 0.18 to 0.65 (average of 0.34) respectively, indicating the potential for a common climate signal of varying strength among sites.

**Figure 4:**
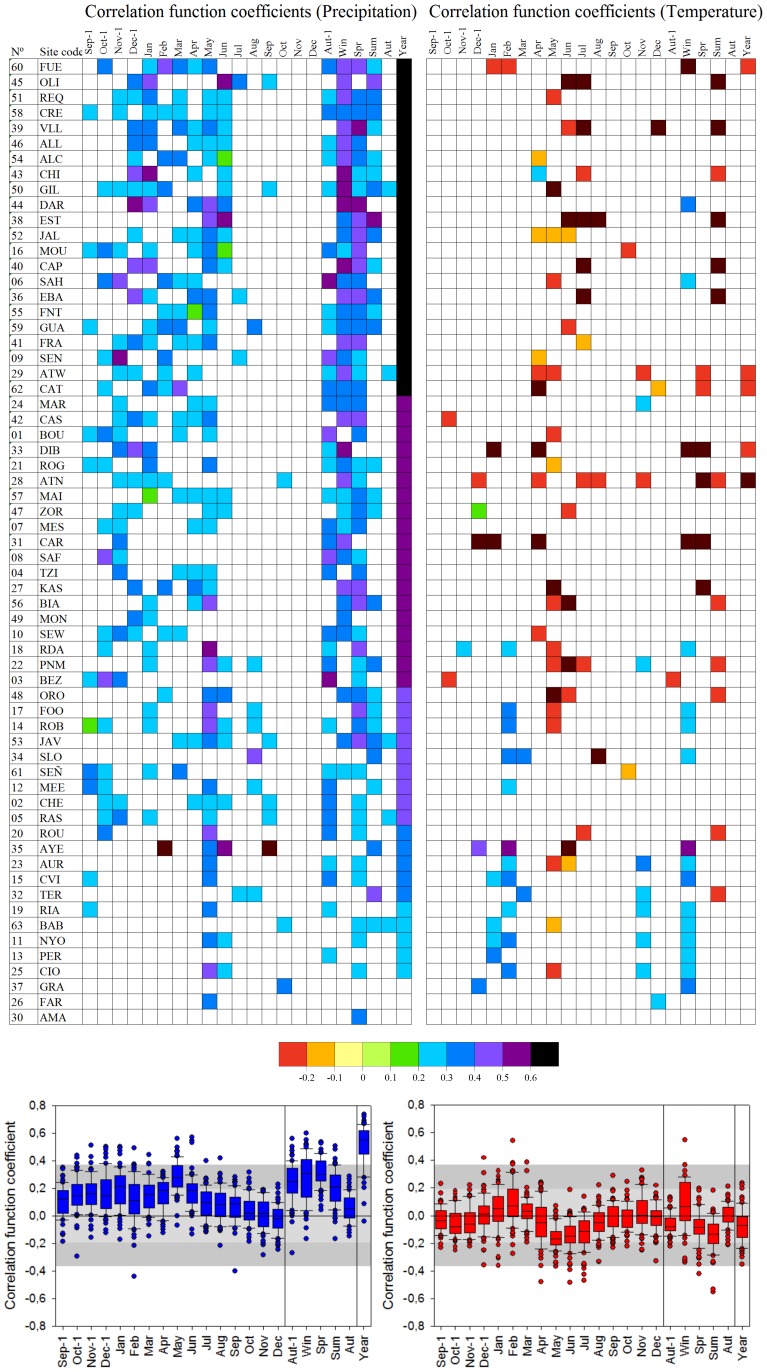
Correlation between the site chronologies of Aleppo pine and climate; monthly, seasonal, and annual precipitation and temperature from September of previous year (Sep-1) to December (Dec) of the current year (top). Only significant values are shown (p<0.05). Box plot showing the variability of correlation coefficients across the chronology network. The central horizontal lines indicate the median values, boxes enclose the central two quartiles, whiskers indicate the 10th and the 90th percentiles and the dots represent the full range of correlation coefficients. Dark and light grey areas indicate significance levels at 99 and 95%, respectively.

The CF analysis showed that ring-width index was primarily related to precipitation and secondarily to temperature. However, a high variability in the relationship between climate and growth was observed across the network (Fig. 4).

Tree-ring index was significantly related to annual total precipitation for all but two chronologies (FAR in France and AMA in Greece). The correlation of tree-ring index to winter temperature was usually significant and positive in colder locations, indicating reduced growth in years with cold winters. In contrast, at three network sites, where conditions are warmer, significant negative correlations indicated reduced growth in years with warm winters.

### Variation in dendroclimatic relationships of Aleppo pine across its distribution

**Figure 5:**
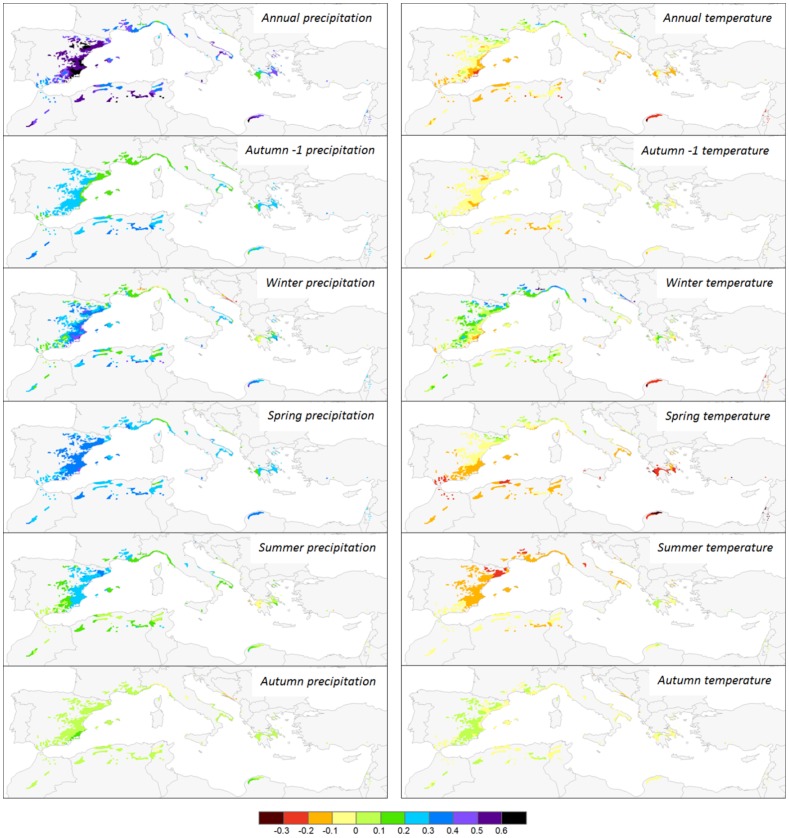
Correlation coefficients across the distribution area of Aleppo pine calculated by using the multiple regression models detailed in Table 2, applied to the values of the significant principal components (PC).

The significant regression equations of CF with the four PCs indicated that dendroclimatic relationships depended on the site position along the climatic gradient of the network (Table 2). This dependence produced a clear and coherent spatial pattern of tree growth in the Mediterranean Basin (Fig. 5). The CFs of annual temperature were inversely related to PC1 indicating that reduced growth associated with warm conditions occurred mainly in the southern part of the species distribution area where temperature was generally higher and precipitation lower than the regional average. Growth limitations due to cold conditions occurred in the northern part of the network where the average temperature was lower and precipitation less limiting than the regional average (Fig. 5). During winter, the area where cold conditions were associated with reduced tree growth was extended to the south. In contrast, in spring and summer reduced tree growth associated with warm conditions occurred in extended areas towards the southern distribution limits of Aleppo pine (Fig. 5).

**Table 2:** Variation of dendroclimatic relationships across climate gradients.

		Intercept		PC1		PC2		PC3		PC4				
		Coef.	Sig	Coef.	Sig	Coef.	Sig	Coef.	Sig	Coef.	Sig	Adjusted r^2^	F	P-value
**Precipitation**	**Sep-1**													ns
	**Oct-1**	0.1432	***	−0.0319	*	−0.0458	*					0.15	3.8	0.008
	**Nov-1**	0.1674	***	0.0364	*			−0.0411	**			0.27	6.6	<0.001
	**Dec-1**	0.1412	***			−0.0728	**			0.0389	*	0.21	5.0	0.001
	**Jan**	0.1633	***			−0.0644	**	0.0354	*			0.21	5.1	0.001
	**Feb**	0.1074	***	0.0692	***							0.17	4.1	0.005
	**Mar**	0.1303	***	0.047	***	−0.0666	***			−0.0245	*	0.44	13.2	<0.001
	**Apr**	0.1462	***	0.0307	*	−0.0531	***					0.3	7.8	<0.001
	**May**	0.2528	***	−0.0623	***			0.0438	***	−0.0218	*	0.45	13.9	<0.001
	**Jun**	0.1371	***	−0.0481	**	−0.0649	***	0.0342	*			0.26	6.5	<0.001
	**Jul**	0.064	***			−0.0604	***					0.19	4.7	0.002
	**Aug**	0.0722	***					0.0474	**			0.21	5.1	0.001
	**Sep**													ns
	**Oct**													ns
	**Nov**			−0.0267	*					−0.0506	***	0.24	5.8	0.001
	**Dec**	−0.0386	**					0.0447	***			0.16	3.9	0.007
	**AUT-1**	0.2373	***					−0.0408	*			0.11	2.9	0.029
	**WIN**	0.2348	***	0.0605	**	−0.0948	***	0.0445	*			0.33	8.7	<0.001
	**SPR**	0.295	***			−0.0527	***			−0.0233	*	0.17	4.2	0.005
	**SUM**	0.1572	***			−0.057	**	0.0662	***			0.28	6.9	<0.001
	**AUT**													ns
	**ANNUAL**	0.4666	***			−0.1166	***	0.0384	*			0.41	11.6	<0.001
**Temperature**	**Sep-1**			0.0292	**							0.13	3.2	0.018
	**Oct-1**	−0.048	***	−0.0262	*	0.0434	**			−0.0286	**	0.31	8.0	<0.001
	**Nov-1**	−0.0397	**	−0.0491	***	0.042	**					0.41	11.7	<0.001
	**Dec-1**			−0.0613	***							0.22	5.3	0.001
	**Jan**	0.0497	**	−0.1033	***			−0.0348	**			0.55	19.7	<0.001
	**Feb**	0.0921	***	−0.0975	***	0.0365	*					0.49	15.9	<0.001
	**Mar**									0.0275	*	0.14	3.5	0.012
	**Apr**	−0.0833	***	−0.1027	***			0.0476	***	0.0242	*	0.57	21.8	<0.001
	**May**													ns
	**Jun**	−0.1125	***	0.0344	**	0.051	***	−0.0374	**			0.27	6.8	<0.001
	**Jul**	−0.0971	***			0.0364	*	−0.0454	**			0.15	3.8	0.009
	**Aug**	−0.0501	***							−0.0299	**	0.2	4.8	0.002
	**Sep**					−0.0443	**			−0.0238	*	0.25	6.1	<0.001
	**Oct**													ns
	**Nov**			−0.0589	***							0.25	6.0	<0.001
	**Dec**					0.0563	***	−0.0243	*			0.19	4.7	0.002
	**AUT-1**	−0.0516	***	−0.0222	**	0.0432	***					0.34	9.1	<0.001
	**WIN**	0.0703	***	−0.1268	***							0.57	21.8	<0.001
	**SPR**	−0.1099	***	−0.0678	***			0.0385	***	0.0285	**	0.51	16.8	<0.001
	**SUM**	−0.1122	***	0.0367	*			−0.0436	**			0.16	4.1	0.006
	**AUT**													ns
	**ANNUAL**	−0.0749	***	−0.0796	***							0.48	15.1	<0.001

Multiple regression models calculated from sets of correlation coefficients from the 63 sites in the dendrochronological network and significant principal components (PC1–PC4) of spatial variability in climate factors.

p<0.05;

p<0.01;

p<0.001.

Similarly, the inverse relationship of CF to PC2 suggests that the positive effects of annual, winter, and spring precipitation on tree growth significantly increased from wetter to drier sites. The influence of winter precipitation also increased along the wet to dry gradient being especially important at sites with high annual temperatures (Fig. 5). PC3 and PC4 were also significantly related to CFs suggesting that the variation in the seasonal distribution of precipitation and temperature across the network influenced the observed dendroclimatic relationships. PC3 was positively related with summer and negatively with winter precipitation while PC4 was positively related with sites with especially high summer temperatures.

## Discussion

### Sensitivity of Aleppo pine to climate variations across its distribution area

The dendrochronological network of 63 sites comprises an essentially complete climatic gradient for Aleppo pine and provides information on how the growth of this species responds to climate variability across its distribution range. In the widely diverse set of climatic conditions, our study demonstrated that Aleppo pine is an excellent species for climate-growth studies. The mean correlation between trees in essentially all chronologies indicated that despite micro-environmental influences, a common pattern of year-to-year variation in ring width exists across all sites. The mean tree-ring sensitivity (the average relative difference in adjacent rings and a measure of climatic responsiveness) was generally higher than observed for other species in the Mediterranean area [41], [42], [43], [44], [45], [46] highlighting the importance of Aleppo pine as a climate proxy for large regional areas. This could be especially important in the Mediterranean region where available climate observations are generally limited to the second half of the 20^th^ century [47]. Additional sampling sites at locations predicted to contain strong climatic signals based on these results would be especially useful to improve reconstruction of past climate. Distribution maps of climate-growth relationships across the distribution range of Aleppo pine may be also especially valuable to predict the response of trees to changing climate patterns.

High sensitivity of a species to climate variations across a wide geographic distribution area or in a wide range of climate conditions as observed here and in other dendrochronological studies suggests that climate change impacts may not be restricted to the ecotone or edge but may occur across the whole distribution range [48], [49]. According to this, our results for Aleppo pine suggest that in the colder sites where low winter temperatures are presently limiting, future increased temperature may have a positive effect on tree growth. However, in the drier and warmer southern Mediterranean, where current positive anomalies in temperature have negative effects on tree growth, conditions for future tree growth will become less favorable as temperatures increase.

### The role of phenotypic plasticity in a species response to climate variability under global warming

The ability of a species to respond through expression of existing genetic variation or phenotypic plasticity to changing environmental conditions may play a decisive role in species persistence or expansion under future global warming [8], [50]. Phenotypic plasticity may be especially important as an adaptive strategy in trees and woody plants since the long generation time of many perennial species implies that the same set of genotypes needs to cope with year-to-year changing environmental conditions [51].

Generally, tree species are considered to express moderate to high plasticity in their responses to environmental stress [52], [53]. Highly variable species that are able to survive in a broader range of environmental conditions are expected to better adjust to future climate conditions [7].

*Pinus halepensis* shows a wide ecological breadth and is adapted to a large range of environmental conditions, abiotic stressors, and perturbations [54]. Several recent studies highlight substantial phenotypic plasticity of *Pinus halepensis* in relation to different anatomical, reproductive and vegetative traits [55], [56], [57], [58], [59], [60], [61], [62], [63], [64], [65], [66], [67], [68], [69], [70].

A substantial plasticity in the annual rhythms of cambial activity of Aleppo pine in response to different climatic conditions has been also well established. Thus, some studies suggest that the cambium is able to maintain activity throughout the whole year when and where climate conditions are favourable [71]. Under other conditions, cambial activity can stop for one to three months during winter, depending on the prevalence of low temperatures [72]. In addition, cambial activity in *Pinus halepensis* can slow down, or even stop, during summer drought [59], [71], [72], [73], [74] and resume later when moisture availability increases in autumn [59], [71], [73], [74], [75], [76]. In other circumstances, the trees can be subjected to “double stress” characterized by two stops in cambial activity - one during winter, caused by low temperatures, and one during the summer triggered by high temperatures and lack of precipitation [73], [74], [77], [78].

However, despite such evidence, plasticity in species responses to climate variability has been poorly explored from a dendrochronological perspective and variability in dendroclimatic response is more often considered as due to environmental noise in the tree-ring signal rather than a consequence of the plastic character of the species.

Geographical variation in climate conditions across the distribution area of a species creates differential selection pressures, and as a consequence population responses to climate variability are likely to vary among genetic provenances as well as through phenotypic plasticity. In this sense, our results highlight significant geographical variations in growth response of Aleppo pine to interannual climate variability suggesting substantial plasticity of growth in response to various climatic conditions. These results do not resolve the source of variability as genetic variation in provenance, phenotypic plasticity or some combination of these or other factors. However, the use of isoenzymes, terpene composition, and other markers has shown that Aleppo pine contains little genetic variability [79], [80] and that intrapopulation genetic variability is generally much higher than interpopulation variability [81]. These findings suggest that phenotypic plasticity has a dominant role in the observed variation in dendroclimatic response across distribution range of Aleppo pine. Then, as current growth responses to inter-annual climate variability vary spatially across existing climate gradients, we expect that future climate-growth relationships will also vary with position along future climate gradients.

### Implications

Historical records of biological responses to climate in situ are perhaps the most dependable data we have to reconstruct past climate conditions, to predict future climate change impacts, and to develop realistic mitigation strategies. The tree-ring record is the most important and widely used source of long-term proxy data as they are available over a wide range of temporal and spatial scales.

However, the use of tree-rings to reconstruct past climate patterns and to forecast of future forest growth within the context of global warming is based on the uniformitarian principle (UP) that assumes that the climate-limiting factors that controlled tree-ring characteristics in the past continue the present and will extend into the future [82]. Under UP, the modeled dendroclimatic relationship is viewed as stationary and consistent through time and the range of climatic variability.

To the contrary, our results show that dendroclimatic relationships significantly vary across species distribution in accordance to underlying climate conditions. Our results also suggest that such variations are likely to be related to the acclimation of trees to the new environmental conditions through a plastic response over decadal time periods. This complicates the simple projection of current relationships into the past or the future. For example, the response to winter temperature change from positive to negative moving from northern (mesic) to southern (drier) sites, with no response at intermediate sites along the thermal gradient. In this sense, the recent warming has a positive effect at the northern limit and a negative effect on the southern portion of the distribution range. At the same time, as summer drought severity increases the response to precipitation disappears, as the tree enters a quiescent and nonreactive state.

Accordingly, the application of UP to dendrochronology may be inappropriate when the dendroclimatic responses are likely to be inherently unstable and climate-dependent as we have shown for Aleppo pine in the Mediterranean basin.

Interestingly, a number of tree-ring studies have addressed changes in tree sensitivity and/or changes in the response of tree growth to climate in coincidence with unprecedented climate warming over the recent decades [83], [84], [85], [86], [87], [88], [89], [90]. The causes of this lack of stability in the dendroclimatic relationship are still not well understood as all potential forcing factors (e.g., changes in climate, atmospheric CO2 concentration, or nitrogen deposition) capable of driving this change covary and obscure the individual impacts [86] but a plausible explanation could be the acclimation of trees to the new environmental conditions through a plastic response over decadal time periods. Our results are related to a limited geographical area and a specific dataset and may have limited application to other species or regions. However, such patterns may occur elsewhere and if so, it may have serious implications by affecting reliability of tree-ring based climate reconstructions.

In addition, climate model validation [91], species distribution models [7], [9] and forecasts of future forest growth within the context of global warming [1], [22], [92], [93], [94] whether or not based on dendrochronology also assumes a uniform response of species to climate variability and change without the competitive advantage conferred by genetic and/or phenotypic plasticity [95]. These assumptions could lead to exaggerate or underestimate species at risk under future climate change.

Accurate prediction of growth of Aleppo pine and other species in response to future climate variability requires an understanding of the plasticity of the response of growth to climate variability and change. A more complex forest dynamics modeling approach considering genetic variation and phenotypic plasticity may contribute to resolve these uncertainties and would result in a more realistic characterization of the biological processes that govern species responses to climatic changes [96], [97], [98]. In this sense, the reliability of old principles and assumptions requires re-examination and complementary theoretical and experimental frameworks should be developed to better understand what trees are telling us in a changing world.

## References

[1] Thuiller W, Araujo MB, Lavorel S (2004) Do we need land-cover data to model species distributions in Europe?. J Biogeogr 31: 353–361.

[2] Peñuelas J, Canadell JG, Ogaya R (2011) Increased water-use efficiency during the 20th century did not translate into enhanced tree growth. Global Ecol Biogeogr 20: 597–608.

[3] Alcamo J, Moreno JM, Nováky B, Bindi M, Corobov R, et al.. (2007) Europe. In: Parry ML, Canziani OF, Palutikof JP, van der Linden PJ, Hanson CE, editors. Climate Change 2007: Impacts, Adaptation and Vulnerability. Contribution of Working Group II to the Fourth Assessment Report of the Intergovernmental Panel on Climate Change. Cambridge, UK. Cambridge University Press, pp. 541–580.

[4] Iverson LR, Prasad AM, Matthews SN, Peters M (2008) Estimating potential habitat for 134 eastern US tree species under six climate scenarios. For Ecol Manage 254: 390–406.

[5] Maiorano L, Cheddadi R, Zimmermann NE, Pellissier L, Petitpierre B, et al (2012) Building the niche through time: using 13,000 years of data to predict the effects of climate change on three tree species in Europe. Global Ecol Biogeogr 22: 302–317.

[6] Nicotra AB, Atkin OK, Bonser SP, Davidson AM, Finnegan EJ, et al (2010) Plant phenotypic plasticity in a changing climate. Trends Plant Sci 15: 684–692. 20970368 10.1016/j.tplants.2010.09.008

[7] Benito-Garzón M, Alía R, Robson TM, Zavala MA (2011) Intra-specific variability and plasticity influence potential tree species distributions under climate change. Global Ecol Biogeogr 20: 766–778.

[8] Matesanz S, Gianoli E, Valladares F (2010) Global change and the evolution of phenotypic plasticity in plants. Ann NY Acad Sci 1206: 35–55. 20860682 10.1111/j.1749-6632.2010.05704.x

[9] Thuiller W, Lavorel S, Araujo MB, Sykes MT, Prentice IC (2005) Climate change threats to plant diversity in Europe. Proc Natl Acad Sci USA 102: 8245. 15919825 10.1073/pnas.0409902102 PMC1140480

[10] Giorgi F, Lionello P (2008) Climate change projections for the Mediterranean region. Global Planet Change 63: 90–104.

[11] Lindner M, Maroschek M, Netherer S, Kremer A, Barbati A, et al (2010) Climate change impacts, adaptive capacity, and vulnerability of European forest ecosystems. For Ecol Manage 259: 698–709.

[12] Peñuelas J, Boada M (2003) A global change-induced biome shift in the Montseny mountains (NE Spain). Global Change Biol 9: 131–140.

[13] Schroter D, Cramer W, Leemans R, Prentice IC, Araujo MB, et al (2005) Ecosystem service supply and vulnerability to global change in Europe. Science 310: 1333–1337. 16254151 10.1126/science.1115233

[14] Christensen JH, Hewitson B, Busuioc A, Chen A, Gao X, et al. (2007) Regional Climate Projections. In: Solomon S, Qin D, Manning M, Chen Z, Marquis M, et al.. editors. Climate Change 2007: The Physical Science Basis. Contribution of Working Group I to the Fourth Assessment Report of the Intergovernmental Panel on Climate Change Cambridge University Press, Cambridge, United Kingdom and New York, NY, USA. pp. 847–940.

[15] Barbéro M, Loisel R, Quézel P, Richardson DM, Romane F (1998) Pines of the Mediterranean Basin. In: Richardson DM, editor. Ecology and Biogeography of Pinus. Cambridge, UK: Cambridge University Press, pp: 153–170.

[16] Quézel P (2000) Taxonomy and biogeography of Mediterranean pines (Pinus halepensis and P. brutia). In: Ne'eman G, Trabaud L, editors. Ecology, Biogeography and Management of Pinus halepensis and P. brutia Forest Ecosystems in the Mediterranean Basin Backhuys, Leiden, The Netherlands. pp. 1–12.

[17] Richardson DM (1998). Ecology and Biogeography of Pinus. Cambridge University Press, Cambridge, UK. pp 3–46.

[18] Ne'eman G, Trabaud L (2000) Biogeography and Management of Pinus halepensis and P. brutia Forest Ecosystems in the Mediterranean Basin. Backhuys, Leiden, The Netherlands.

[19] Touchan R, Hughes MK (1999) Dendrochronology in Jordan. J Arid Environ 42: 291–303.

[20] Papadopoulos A, Serre-Bachet F, Tessier L (2001) Tree ring to climate relationships of Aleppo pine (Pinus halepensis Mill.) in Greece. Ecologia Mediterranea 27: 89–98.

[21] Rathgeber C, Nicault A, Kaplan JO, Guiot J (2003) Using a biogeochemistry model in simulating forests productivity responses to climatic change and [CO2] increase: example of Pinus halepensis in Provence (south-east France). Ecol Modell 166: 239–255.

[22] Rathgeber C, Nicault A, Guiot J, Keller T, Guibal F, et al (2000) Simulated responses of Pinus halepensis forest productivity to climatic change and CO2 increase using a statistical model. Global Planet Change 26: 405–421.

[23] Touchan R, Xoplaki E, Funkhouser G, Luterbacher J, Hughes MK, et al (2005) Reconstructions of spring/summer precipitation for the Eastern Mediterranean from tree-ring widths and its connection to large-scale atmospheric circulation. Clim Dyn 25: 75–98.

[24] Sarris D, Christodoulakis D, Korner C (2007) Recent decline in precipitation and tree growth in the eastern Mediterranean. Global Change Biol 13: 1187–1200.

[25] de Luis M, Novak K, Cufar K, Raventos J (2009) Size mediated climate-growth relationships in Pinus halepensis and Pinus pinea. Trees Struct Funct 23: 1065–1073.

[26] Pasho E, Camarero JJ, de Luis M, Vicente-Serrano SM (2011) Spatial variability in large-scale and regional atmospheric drivers of Pinus halepensis growth in eastern Spain. Agric For Meteorol 151: 1106–1119.

[27] Attolini MR, Calvani F, Galli M, Nanni T, Ruggiero L, et al (1990) The relationship between climatic variables and wood structure in Pinus halepensis Mill. Theor Appl Climatol 41: 121–127.

[28] Olivar J, Bogino S, Spiecker H, Bravo F (2012) Climate impact on growth dynamic and intra-annual density fluctuations in Aleppo pine (Pinus halepensis) trees of different crown classes. Dendrochronologia 30: 35–47.

[29] Novak K, de Luis, M, Raventós J, Čufar K (2013) Climatic signals in tree-ring widths and wood structure of Pinus halepensis in contrasted environmental conditions. Trees Struct Funct 27: 927–936.

[30] Critchfield WB, Little EL (1966) Geographic distribution of the pines of the World. USDA For Serv Misc Publ 991.

[31] Mitchell TD, Carter TR, Jones PD, Hulme M, New M (2004) A comprehensive set of high-resolution grids of monthly climate for Europe and the globe: the observed record (1901–2000) and 16 scenarios (2001–2100). Tyndall Center Working Paper 55: 1–30.

[32] Mitchell TD, Jones PD (2005) An improved method of constructing a database of monthly climate observations and associated high-resolution grids. Int J Climatol 25: 693–712.

[33] Jolliffe IT (1986) Principal Component Analysis. Springer-Verlag, pp. 487. DOI: 10.1007/b98835. ISBN 978-0-387-95442-4.

[34] Richman MB (1986) Rotation of principal components. J Clim 6: 29–35.

[35] Kaiser HF (1992) On Cliff's formula, the Kaiser-Guttman rule, and the number of factors. Perceptual and Motor Skills 74: 595–598.

[36] Speer JH (2010) Fundamentals of Tree-Ring Research. University of Arizona Press, Tucson, Arizona, USA, 333 pp.

[37] Cook ER, Kairiukstis LA (1990) Methods of Dendrochronology. Kluwer Academic Publishers, Dordrecht/Boston/London. 394 pp.

[38] Cook ER (1985) A time series analysis approach to tree-ring standardization. PhD dissertation, University of Arizona, Tucson, Arizona. 171 pp.

[39] Wigley TML, Briffa KR, Jones PD (1984) On the average value of correlated time-series, with applications in dendroclimatology and hydrometeorology. J Clim Appl Meteorol 23: 201–213.

[40] Biondi F, Waikul K (2004) DENDROCLIM2002: A C++ program for statistical calibration of climate signals in tree-ring chronologies. Comput Geosci 30: 303–311.

[41] Andreu L, Gutierrez E, Macias M, Ribas M, Bosch O, et al (2007) Climate increases regional tree-growth variability in Iberian pine forests. Global Change Biol 13: 804–815.

[42] Buntgen U, Frank D, Trouet V, Esper J (2010) Diverse climate sensitivity of Mediterranean tree-ring width and density. Trees Struct Funct 24: 261–273.

[43] Carrer M, Nola P, Motta R, Urbinati C (2010) Contrasting tree-ring growth to climate responses of Abies alba toward the southern limit of its distribution area. Oikos 119: 1515–1525.

[44] Lebourgeois F, Rathgeber CBK, Ulrich E (2010) Sensitivity of French temperate coniferous forests to climate variability and extreme events (Abies alba, Picea abies and Pinus sylvestris). J Veg Sci 21: 364–376.

[45] Mérian P, Lebourgeois F (2011) Size-mediated climate–growth relationships in temperate forests: A multi-species analysis. For Ecol Manage 261: 1382–1391.

[46] Babst F, Poulter B, Trouet V, Tan K, Neuwirth B, et al (2013) Site- and species-specific responses of forest growth to climate across the European continent. Global Ecol Biogeogr 22: 706–717.

[47] González-Hidalgo JC, Brunetti M, de Luis M (2011) A new tool for monthly precipitation analysis in Spain: MOPREDAS database (monthly precipitation trends December 1945–November 2005). Int J Climatol 31: 715–731.

[48] Hamann A, Wang TL (2005) Models of climatic normals for genecology and climate change studies in British Columbia. Agric For Meteorol 128: 211–221.

[49] Chen PY, Welsh C, Hamann A (2010) Geographic variation in growth response of Douglas-fir to interannual climate variability and projected climate change. Global Change Biol 16: 3374–3385.

[50] Matesanz S, Valladares F (2013) Ecological and evolutionary responses of Mediterranean plants to global change. Environ Exp Bot DOI:http://dx.doi.org/10.1016/j.envexpbot.2013.09.004

[51] Willson MF (1983) Plant Reproductive Ecology. John Wiley & Sons, New York, USA.

[52] Wagner F, Below R, de Klerk P, Dilcher DL, Joosten H, et al (1996) A natural experiment on plant acclimation: lifetime stomatal frequency response of an individual tree to annual atmospheric CO2 increase. Proc Natl Acad Sci USA 93: 11705–11708. 11607712 10.1073/pnas.93.21.11705 PMC38122

[53] Climent JM, Aranda I, Alonso J, Pardos JA, Gil L (2006) Developmental constraints limit the response of Canary Island pine seedlings to combined shade and drought. For Ecol Manage 231: 164–168.

[54] Ne'eman G, Goubitz S, Nathan R (2004) Reproductive traits of Pinus halepensis in the light of fire – a critical review. Plant Ecol 171: 69–79.

[55] Baquedano FJ, Valladares F, Castillo FJ (2008) Phenotypic plasticity blurs ecotypic divergence in the response of Quercus coccifera and Pinus halepensis to water stress. Eur J Forest Res 127: 495–506.

[56] Chambel MR, Climent J, Alía R (2007) Divergence among species and populations of Mediterranean pines in biomass allocation of seedlings grown under two watering regimes. Ann Forest Sci 64: 87–97.

[57] Climent J, Prada MA, Calama R, Chambel R, Sánchez de Ron D, et al (2008) To grow or to seed: ecotypic variation in reproductive allocation and cone production by young female Aleppo pine (Pinus halepensis, pinaceae). Am J Bot 95: 1–10. 21632409 10.3732/ajb.2007354

[58] Cuesta B, Villar-Salvador P, Puértolas J, Jacobs DF, Rey Benayas JM (2010) Why do large, nitrogen rich seedlings better resist stressful transplanting conditions? A physiological analysis in two functionally contrasting Mediterranean forest species. For Ecol Manage 260: 71–78.

[59] de Luis M, Novak K, Raventós J, Gričar J, Prislan P, et al (2011) Cambial activity, wood formation and sapling survival of Pinus halepensis exposed to different irrigation regimes. For Ecol Manage 262: 1630–1638.

[60] Froux F, Ducrey M, Epron D, Dreyer E (2004) Seasonal variations and acclimation potential of the thermostability of photochemistry in four Mediterranean conifers. Ann Forest Sci 61: 235–241.

[61] García-Esteban L, Martín JA, de Palacios P, García Fernández F, López R (2010) Adaptive anatomy of Pinus halepensis trees from different Mediterranean environments in Spain. Trees Struct Funct 24: 19–30.

[62] Michelozzi M, Loreto F, Colom R, Rossi F, Calamassi R (2011) Drought responses in Aleppo pine seedlings from two wild provenances with different climatic features. Photosynthetica 49: 564–572.

[63] Monnier Y, Vila B, Montès N, Bousquet-Mélou A, Prévosto B, et al (2011) Fertilization and allelopathy modify Pinus halepensis saplings crown acclimation to shade. Trees Struct Funct 25: 497–507.

[64] Monnier Y, Bousquet-Mélou A, Vila B, Prévosto B, Fernandez C (2013) How nutrient availability influences acclimation to shade of two (pioneer and late-successional) Mediterranean tree species?. Eur J Forest Res 132: 325–333.

[65] Pardos M, Climent J, Gil L, Pardos JA (2003) Shoot growth components and flowering phenology in grafted Pinus halepensis Mill. Trees Struct Funct 17: 442–450.

[66] Santos del Blanco L, Zas R, Notivol E, Chambel MR, Majada J, et al (2010) Variation of early reproductive allocation in multi-site genetic trials of Maritime pine and Aleppo pine. Forest Systems 19: 381–392.

[67] Santos del Blanco L, Bonser SP, Valladares F, Chambel MR, Climent J (2013) Plasticity in reproduction and growth among 52 range-wide populations of a Mediterranean conifer: Adaptive responses to environmental stress. J Evolution Biol 26: 1912–1924. 10.1111/jeb.12187 23944274

[68] Sardans J, Rodà F, Peñuelas J (2004) Phosphorus limitation and competitive capacities of Pinus halepensis and Quercus ilex subsp. rotundifolia on different soils. Plant Ecol 174: 305–317.

[69] Voltas J, Chambel M, Prada M, Ferrio J (2008) Climate-related variability in carbon and oxygen stable isotopes among populations of Aleppo pine grown in common-garden tests. Trees Struct Funct 22: 759–769.

[70] Zavala MA, Espelta JM, Caspersen J, Retana J (2011) Interspecific differences in sapling performance with respect to light and aridity gradients in mediterranean pine-oak forests: Implications for species coexistence. Can J Forest Res 41: 1432–1444.

[71] Liphschitz N, Lev-Yadun S, Rosen E, Waisel Y (1984) The Annual Rhythm of Activity of the Lateral Meristems (Cambium and Phellogen) in Pinus halepensis Mill and Pinus pinea L. IAWA Bulletin 5: 263–274.

[72] Gindel I (1967) Cambial activity as a function of the intensity of transpiration in Pinus halepensis Mill. Proc XVI IUFRO Congr. München 1967, vol IV, sect.23: 188–206.

[73] de Luis M, Gričar J, Čufar K, Raventós J (2007) Seasonal dynamics of wood formation in Pinus halepensis from dry and semi-arid ecosystems in Spain. IAWA Journal 28: 389–404.

[74] de Luis M, Novak K, Raventos J, Gricar J, Prislan P, et al (2011) Climate factors promoting intra-annual density fluctuations in Aleppo pine (Pinus halepensis) from semiarid sites. Dendrochronologia 29: 163–169.

[75] Camarero JJ, Olano JM, Parras A (2010) Plastic bimodal xylogenesis in conifers from continental Mediterranean climates. New Phytol 185: 471–480. 19895415 10.1111/j.1469-8137.2009.03073.x

[76] Novak K, Saz Sánchez MA, Čufar K, Raventós J, de Luis M (2013) Age, climate and intra-annual density fluctuations in Pinus halepensis in Spain. IAWA Journal 34: 459–474.

[77] Cherubini P, Gartner BL, Tognetti R, Braker OU, Schoch W, et al (2003) Identification, measurement and interpretation of tree rings in woody species from Mediterranean climates. Biol Rev 78: 119–148. 12620063 10.1017/s1464793102006000

[78] Lev-Yadun S (2000) Wood structure and the ecology of annual growth ring formation in Pinus halepensis and P. brutia. In: Ecology, Biogeography and Management of Pinus halepensis and P. brutia Forest Ecosystems in the Mediterranean Basin (eds Ne'eman G, Trabaud L), pp. 67–78. Backhuys Publishers, Leiden, The Netherlands.

[79] Schiller G, Gonkle MT, Grunwald C (1986) Local differentiation among Mediterranean populations of Aleppo pine in their isoenzymes. Silvae Genet 35: 11–19.

[80] Soto A, Robledo-Arnuncio JJ, González-Martínez SC, Smouse PE, Alia R (2010) Climatic niche and neutral genetic diversity of the six Iberian pine species: a retrospective and prospective view. Mol Ecol 19: 1396–1409. 20196810 10.1111/j.1365-294X.2010.04571.x

[81] Gómez A, Alia R, Bueno MA (2001) Genetic diversity of Pinus halepensis Mill. populations detected by RAPD loci. Ann For Sci 58: 869–875.

[82] Fritts HC (1976) Tree Rings and Climate. Academic Press, New York.

[83] Briffa KR, Schweingruber FH, Jones PD, Osborn TJ, Shiyatov SG, et al (1998) Reduced sensitivity of recent tree-growth to temperature at high northern latitudes. Nature 391: 678–682.

[84] Carrer M, Urbinati C (2004) Age-dependent tree-ring growth responses to climate in Larix decidua and Pinus cembra. Ecology 85: 730–740.

[85] Buntgen U, Frank D, Wilson R, Carrer M, Urbinati C, et al (2008) Testing for tree-ring divergence in the European Alps. Glob Change Biol 14: 2443–2453.

[86] D'Arrigo R, Wilson R, Liepert B, Cherubini P (2008) On the ‘Divergence Problem’ in Northern Forests: A review of the tree-ring evidence and possible causes. Glob Planet Change 60: 289–305.

[87] Carrer M, Nola P, Eduard JL, Motta R, Urbinati C (2007) Regional variability of climate-growth relationships in Pinus cembra high elevation forests in the Alps. J Ecol 95: 1072–1083.

[88] di Filippo A, Biondi F, Cufar K, de Luis M, Grabner M, et al (2007) Bioclimatology of beech (Fagus sylvatica L.) in the Eastern Alps: spatial and altitudinal climatic signals identified through a tree-ring network. J Biogeograph 34: 1873–1892.

[89] Wilmking M, Singh J (2008) Eliminating the “divergence problem” at Alaska's northern treeline. Climate Past Discuss 4: 741–759.

[90] Esper J, Frank D, Buntgen U, Verstege A, Hantemirov RM, et al (2010) Trends and uncertainties in Siberian indicators of 20th century warming. Glob Change Biol 16: 386–398.

[91] Randall DA, Wood RA, Bony S, Colman R, Fichefet T, et al. (2007) Climate models and their evaluation. In: Solomon S, Qin D, Manning M, Chen Z, Marquis M, Averyt KB, et al.. editors. Climate Change 2007: The Physical Science Basis. Contribution of Working Group I to the Fourth Assessment Report of the Intergovernmental Panel on Climate Change (Eds.) Cambridge University Press, Cambridge, United Kingdom and New York, NY, USA.

[92] Thuiller W, Lavorel S, Sykes MT, Araujo MB (2006) Using niche-based modelling to assess the impact of climate change on tree functional diversity in Europe. Divers Distrib 12: 49–60.

[93] Keenan T, Serra Diaz JM, Lloret F, Ninyerola M, Sabaté S (2011) Predicting the future of forests in the Mediterranean under climate change, with niche- and process-based models: CO2 matters!. Glob Change Biol 17: 565–579.

[94] Serra Diaz JM, Keenan TF, Ninyerola M, Sabaté S, Gracia C, et al (2013) Geographical patterns of congruence and incongruence between correlative species distribution models and a process-based ecophysiological growth model. J Biogeogr 40: 1928–1938.

[95] Rodder D, Lotters S (2009) Niche shift versus niche conservatism? Climatic characteristics of the native and invasive ranges of the Mediterranean house gecko (Hemidactylus turcicus). Global Ecol Biogeogr 18: 674–687.

[96] Pearson RG, Dawson TP (2003) Predicting the impacts of climate change on the distribution of species: are bioclimate envelope models useful? Global Ecol Biogeogr 12: 361–371.

[97] Montoya D, Purves DW, Urbieta IR, Zavala MA (2009) Do species distribution models explain spatial structure within tree species ranges?. Global Ecol Biogeogr 18: 662–673.

[98] Roberts DR, Hamann A (2012) Predicting potential climate change impacts with bioclimate envelope models: a palaeoecological perspective. Global Ecol Biogeogr 21: 121–133.

