# Correction: Plasticity in Dendroclimatic Response across the Distribution Range of Aleppo Pine (*Pinus halepensis*)

**DOI:** 10.1371/annotation/5e1bcd37-a7f8-4f8b-b621-e58bb3caea90

**Published:** 2014-01-23

**Authors:** Martin de Luis, Katarina Čufar, Alfredo Di Filippo, Klemen Novak, Andreas Papadopoulos, Gianluca Piovesan, Cyrille B. K. Rathgeber, José Raventós, Miguel Angel Saz, Kevin T. Smith

Affiliation number 8 for the last author, Kevin T. Smith, is incorrect. The correct affiliation is: Northern Research Station, USDA Forest Service, Durham, New Hampshire, United States of America.

